# The Value of the Stemness Index in Ovarian Cancer Prognosis

**DOI:** 10.3390/genes13060993

**Published:** 2022-05-31

**Authors:** Hongjun Yuan, Qian Yu, Jianyu Pang, Yongzhi Chen, Miaomiao Sheng, Wenru Tang

**Affiliations:** Laboratory of Molecular Genetics of Aging & Tumor, Medical School, Kunming University of Science and Technology, Kunming 650032, China; yuanttx@163.com (H.Y.); mr_yuq@163.com (Q.Y.); jianyu_0898@163.com (J.P.); cyz1206414925@163.com (Y.C.); miaomiaosheng156@163.com (M.S.)

**Keywords:** ovarian cancer, stemness index, prognostic, immune infiltration

## Abstract

Ovarian cancer (OC) is one of the most common gynecological malignancies. It is associated with a difficult diagnosis and poor prognosis. Our study aimed to analyze tumor stemness to determine the prognosis feature of patients with OC. At this job, we selected the gene expression and the clinical profiles of patients with OC in the TCGA database. We calculated the stemness index of each patient using the one-class logistic regression (OCLR) algorithm and performed correlation analysis with immune infiltration. We used consensus clustering methods to classify OC patients into different stemness subtypes and compared the differences in immune infiltration between them. Finally, we established a prognostic signature by Cox and LASSO regression analysis. We found a significant negative correlation between a high stemness index and immune score. Pathway analysis indicated that the differentially expressed genes (DEGs) from the low- and high-mRNAsi groups were enriched in multiple functions and pathways, such as protein digestion and absorption, the PI3K-Akt signaling pathway, and the TGF-β signaling pathway. By consensus cluster analysis, patients with OC were split into two stemness subtypes, with subtype II having a better prognosis and higher immune infiltration. Furthermore, we identified 11 key genes to construct the prognostic signature for patients with OC. Among these genes, the expression levels of nine, including *SFRP2*, *MFAP4*, *CCDC80*, *COL16A1*, *DUSP1*, *VSTM2L*, *TGFBI*, *PXDN*, and *GAS1*, were increased in the high-risk group. The analysis of the KM and ROC curves indicated that this prognostic signature had a great survival prediction ability and could independently predict the prognosis for patients with OC. We established a stemness index-related risk prognostic module for OC, which has prognostic-independent capabilities and is expected to improve the diagnosis and treatment of patients with OC.

## 1. Introduction

Ovarian cancer (OC) involves thorny tumors with high malignancy and a difficult diagnosis. It poses a health threat to women worldwide [1]. OC remains the deadliest gynecological tumor, despite recent improvements in survival times. Compared to other gynecological cancers, the 5-year survival rate of OC is less than 40% [2]. Drug resistance and high rates of recurrence are the main causes of this poor prognosis [3]. Currently, surgical debulking and chemotherapy are still the main treatment modalities for OC [4]. Moreover, immunotherapy is also applied to OC treatment owing to its widespread application [5].

Cancer stem cells (CSCs) belong to a type of malignant tumor cells with stemness characteristics that have the potential for differentiation and self-renewal [6]. CSCs have been studied in many solid tumors, including breast cancer [7], lung cancer [8], and OC [9]. These cells are thought to be responsible for the spread and metastasis of tumors [7,10]. The stemness characteristic of CSCs is considered have an important effect on the resistance to tumor chemotherapy and could be a potential therapeutic target [11]. Many studies have found that the abundance of CSCs in breast cancer and gliomas is negatively correlated with treatment [12], indicating a relation to patients [6]. In melanoma, a subset of CSCs evade the immune system by negatively regulating the expression of T cell function and secreting immunosuppressive factors, which may be involved in tumor recurrence [13]. CSCs also promote OC migration and resistance to therapy. Platinum-based anticancer drugs cannot eliminate CSCs [14,15], which could lead to metastasis and OC recurrence [9]. Increasing evidence suggests that CSC elimination will suppress OC growth and recurrence [16,17]. Therefore, investigating the role of CSCs in OC may improve clinical results.

To quantify CSCs’ characteristics, Malta et al. [18] identified and quantified CSCs’ characteristics based on the OCLR algorithm and proposed mRNA expression to quantify the stemness index (mRNAsi). In this job, we explored the role of the stemness index in OC and identified the prognostic capacity of the stemness index for patients with OC. We counted the stemness index and immune score for all OC samples and analyzed the association between mRNAsi and immune infiltration. Then, we obtained the DEGs between the high- and low-mRNAsi groups and performed a functional enrichment analysis. Based on these DEGs, we classified OC patients into two stemness subtypes using the consensus clustering method. The two stemness subtypes showed significant differences in immune infiltration, and subtype II showed a better prognosis (*p* < 0.05). Finally, we established a prognostic signature for OC and demonstrated that this signature was independent.

## 2. Materials and Methods

### 2.1. Data Obtained

The gene expression and corresponding clinical characteristic profiles of OC patients were obtained from the University of California Santa Cruz (UCSC) Xena database. The RNA-seq data were measured by fragments per kilobase of transcript per million mapped reads and normalized by log2. Patients without complete clinical information were removed. The expression data of the patients with OC (OV-AU) was obtained from the International Cancer Genome Consortium (ICGC) database as the validation cohort.

### 2.2. Calculation of the Stemness Index and Immune Score

In this research, we downloaded the stem cell expression profiles (syn2701943) in the Progenitor Cell Biology Consortium database and used the OCLR algorithm to count the mRNAsi of each patient. The mRNAsi value is scaled 0–1 accordingly; the higher the value, the higher the activity of the cancer stem cells. Patients were placed into the high- or low-mRNAsi groups using the median mRNAsi. Then, we obtained the immune scores, stromal scores, and ESTIMATE scores of all patients using the ESTIMATE algorithm. Patients were also split into high- or low-immunity groups using the median immune score.

### 2.3. Correlation Analysis of the Stemness Index and Immune Infiltration

To analyze the correlation between mRNAsi and immune infiltration, we applied the ssGSEA and CIBERSORT algorithms [19]. We collected a set of 28 immune-related genes [20] and used ssGSEA to calculate the rank value of each gene from the expression profile and quantified the enrichment score of each gene. Single immune-related genes in each sample can be used to determine the immune cell activity or immune pathway. The CIBERSORT method provides a set of gene signatures for 22 tumor-infiltrating immune cell fractions, including naive B cell, memory B cell, CD4+ resting memory T cell, etc.

### 2.4. Differentially Expressed and Functional Enrichment Analyses

The “limma” function was applied to identify the DEGs from the two mRNAsi groups. The selection criteria for DEGs were an FDR < 0.05 and |log2 fold change (FC)| > 1. To analyze the possible functions and pathways involved in these DEGs, we used the “clusterProfiler” package for functional annotation. Gene Ontology (GO) was performed for functional annotation and Kyoto Encyclopedia of Genes and Genomes (KEGG) were performed to assess related pathways.

### 2.5. Data-Obtained Identification of OC’s Stemness-Related Molecular Subtypes

We performed a consensus cluster analysis with the “ConsensusClusterPlus” package according to DEGs and identified different stemness subtypes. The number of replicates of the cluster analysis was set to 100, and 80% of the samples were used for each replicate. We used the consensus heatmap and the cumulative distribution function (CDF) to select the optimal number of clusters. The gene set variation analysis (GSVA) was performed to explore the pathways in the stemness subtypes using the package “GSVA”. The KEGG pathway profile was downloaded from the molecular signatures database (MSigDB).

### 2.6. Evaluation of the Relationship between Stemness Subtypes and Immune Infiltration

To assess the connection between immune infiltration and stemness subtype, we first compared the correlation between the immune score and stemness subtype, and the level of immune infiltration between different subtypes. Next, we compared the difference in the tumor mutation burden (TMB) value between the different stemness subtypes. We also compared the six immune checkpoint expression levels in different stemness subtypes, including *PDCD1*, *CD80*, *CD274*, *PDCD1LG2*, *CTLA4*, and *CD86*.

### 2.7. Construction and Validation of the Prognostic Signature

First, we obtained the genes related to prognosis based on DEGs using the univariate Cox regression analysis. Genes with significance were chosen for the subsequent analysis. The least absolute shrinkage and selection operator (LASSO) regression analysis was used to determine the best suitable genes. Finally, the remaining genes and corresponding coefficients were retained to establish the risk prognostic signature, which was: Risk score = $\sum_{i}^{n}\mathrm{Coef}\left(i\right)\times \mathrm{Exp}\left(i\right)$ (*n*: the amount of genes; *i*: gene; Coef: coefficients; Exp: gene expression level).

The patients in the TCGA cohort were assigned to high- or low-risk groups using the median risk score. Then, we used the survival information from the two risk groups to plot the Kaplan–Meier (KM) survival curve. We performed receiver operating characteristic (ROC) analysis, and the area under the ROC curve (AUC) was used to evaluate the signature value. ICGC data was used as a validation cohort.

### 2.8. Identification of Prognostic Factors and Nomogram Construction

To explore whether this risk signature has the ability to independently prognosticate, we extracted clinical characteristics, including age and clinical stage. We evaluated these variables in combination with risk scores using Cox regression analysis. We constructed prognostic nomograms using independent prognostic factors identified by Cox regression analysis and tested the predictive accuracy of the nomogram using calibration plots.

### 2.9. Statistical Analysis

All statistical analyses were performed using R software (version 4.1.1). We used the log-rank test to calibrate the difference in the survival analysis. The Cox regression analysis was applied to calculate the connection between survival outcomes and gene expression. *p* < 0.05 was considered statistically significant.

## 3. Results

### 3.1. Correlation between the Stemness Index and Clinical Characteristics

**Figure 1:**
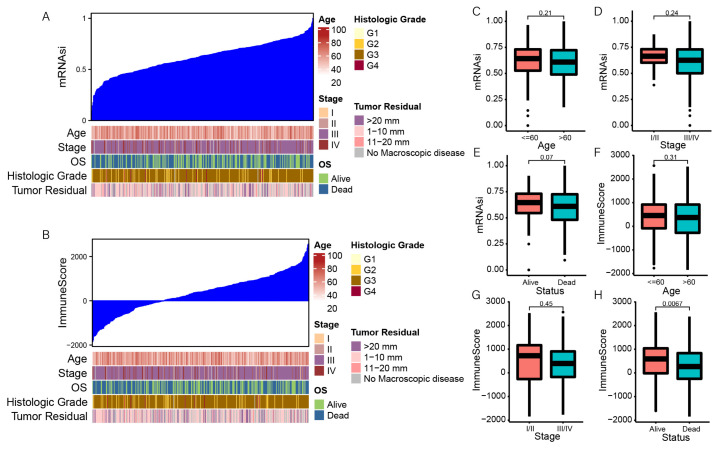
The clinical characteristics associated with the mRNAsi and immune score in OC patients. (**A**) The general picture of the association between mRNAsi and the clinical features. (**B**) The general picture of the association between the immune score and the clinical features. (**C**–**E**) The correlation between mRNAsi and age, clinical stage, and clinical status. (**F***–***H**) The correlation between immune score and age, clinical stage, and clinical status.

To investigate the correlation between mRNAsi and clinical characteristics of OC, we calculated the stemness index and immune score of 379 OC patients using the OCLR and ESTIMATE algorithms. We then ranked patients to explore the relationship between mRNAsi and clinical characteristics (Figure 1A,B). We divided all patients into different groups according to the clinical characteristics and then compared the mRNAsi expression in various clinical characteristics. Association analysis showed that mRNAsi did not significantly differ by age and clinical stage (Figure 1C,D). We found that the value of mRNAsi in the survival group was higher than that in the group that died, but this was insignificant (Figure 1E). However, patients in the survival group had significantly higher immune scores than those in the group that died (*p* = 0.0067; Figure 1H). There were no significant differences in the immune scores by age or clinical stage (Figure 1F,G).

### 3.2. Correlation between mRNAsi and Immune Infiltration

**Figure 2:**
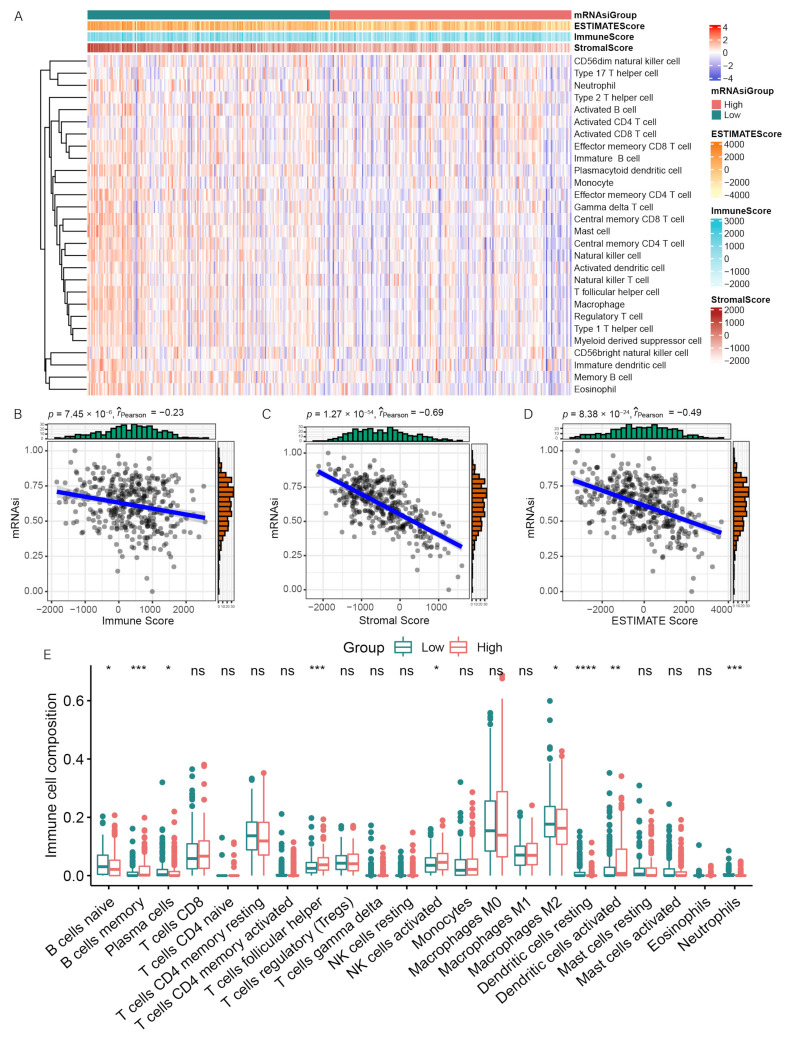
The immune features of OC that are associated with mRNAsi. (**A**) Correlation between mRNAsi and immune infiltration. (**B**–**D**) Correlation between mRNAsi and the immune score, stromal score, and ESTIMATE score. The blue line is the regression line of mRNAsi and other scores. (**E**) Comparisons of the abundances of 22 immune cells in 2 mRNAsi groups. * *p* < 0.05; ** *p* < 0.01; *** *p* < 0.001, **** *p* < 0.0001.

Considering the important influence of immune infiltration in tumor treatment, and the differences in the immune scores between clinical outcomes, we investigated the correlation between mRNAsi and immune infiltration. The enrichment levels of 28 immune-related signatures were quantified using the ssGSEA method, reflecting the immune activity. The result showed that the immune activity in the low-mRNAsi group was higher than that in the high-mRNAsi group (Figure 2A). The correlation analysis showed that mRNAsi was significantly negatively correlated with the immune score, stromal score, and ESTIMATE score (*p* < 0.01), which indicated that the immune cell infiltration levels decrease with elevated OC stemness (Figure 2B–D). We then quantified the abundances of the 22 immune cell types in the 2 mRNAsi groups using the CIBERSORT algorithm. We found that the mRNAsi was significantly positively correlated with B cell memory, T cell follicular helper cells, activated NK cells, and activated dendritic cells. mRNAsi was significantly negatively correlated with plasma cells, naive B cells, M2 macrophages, dendritic cells, and neutrophils (Figure 2E).

### 3.3. Differentially Expressed and Functional Enrichment Analyses

**Figure 3:**
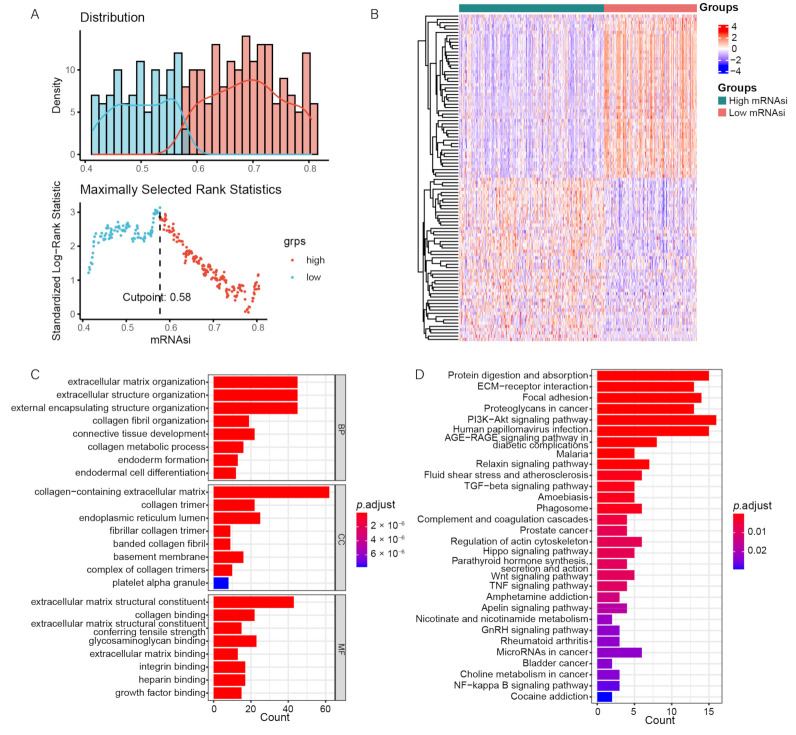
Differential expression analysis and functional enrichment analysis. (**A**) We determined 0.58 as the optimal grouping value. (**B**) The heatmap reflects the expression levels of DEGs. (**C**) The GO functional annotation analysis. (**D**) The KEGG pathway enrichment analysis.

Next, we attempted to explore the differences in the functional annotation and pathway enrichment analysis between the groups categorized by miRNA. Since there were no significant differences between samples grouped by the median mRNAsi value, we determined an optimal cutoff of mRNAsi = 0.58 based on the results of the “survminer” analysis (Figure 3A) to obtain a more reasonable grouping. We reclassified 379 OC patients into the high-mRNAsi group (*n* = 231) or the low-mRNAsi group (*n* = 148). We then performed a differential expression analysis in the 2 mRNAsi groups and identified 156 DEGs (Figure 3B).

We performed DAVID using these DEGs to investigate their possible biological functions. According to the results of the functional enrichment analysis, we found more than 50 enriched biological processes, including extracellular structure organization, extracellular matrix organization, and external encapsulating structure organization; 31 enriched cellular components, including fibrillar collagen trimer, collagen trimer, collagen-containing extracellular matrix, and endoplasmic reticulum lumen; 42 enriched molecular functions, including extracellular matrix structural constituent, collagen binding, and extracellular matrix structural constituent conferring tensile strength (Figure 3C); and 28 enriched KEGG pathways, including the PI3K-Akt signaling pathway, Wnt signaling pathway, and TGF-β signaling pathway (Figure 3D). These results indicate that these DEGs are associated with the tumor signaling pathway and may regulate tumor progression.

### 3.4. Identification of Two Stemness Subtypes with Distinct Characteristics

**Figure 4:**
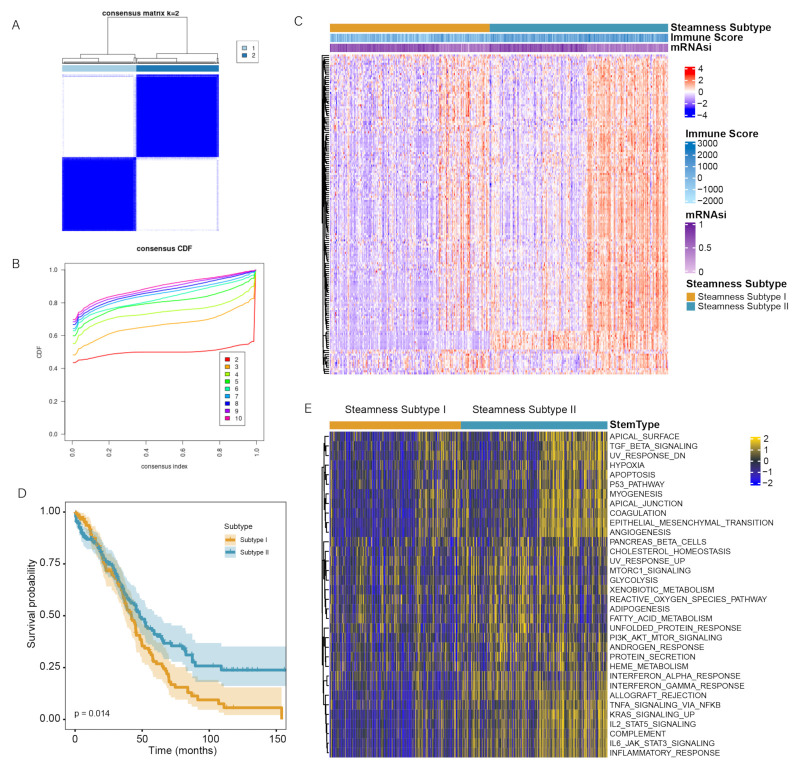
Identification of the two stemness subtypes. (**A**) Consensus clustering heatmap when k = 2. (**B**) CDF curves of the consensus score from k  =  2 to 10. (**C**) The heatmap of DEGs between the two subtypes. (**D**) KM curve analysis exhibited that the patients in stemness subtype II have significantly better OS. (**E**) Heatmap showed 33 differentially enriched pathways between the 2 subtypes.

To analyze the association between mRNAsi and OC subtypes, we used the consensus clustering method to explore a novel classification of OC in the TCGA cohort. According to the consensus heatmap and the CDF curve, the intergroup connections were the lowest and the intragroup connections were the highest when k = 2 (Figure 4A,B, Supplementary Table S1). Therefore, 379 patients with OC were classified into 2 stemness subgroups (Figure 4C), including stemness subtype I (201 patients, 53.2%) and stemness subtype II (178 patients, 47.8%). The demographic information between the two stemness subtypes is shown in Supplementary Table S2. Survival analysis indicated that patients with OC in the stemness subtype II had a better OS time than those in the stemness subtype I (*p*  =  0.014, Figure 4D). The median OS time of the patients in the stemness subtype II was longer than that in the stemness subtype I.

Subsequently, we performed differential expression analysis on the two subtypes and performed GSVA to analyze the molecular pathways and underlying functions associated with the stemness subtype. Finally, we identified 33 significantly enriched pathways that were positively related to the stemness subtype II (Figure 4E). The results revealed that stemness subtype II tumors were primarily related to tumorigenesis (e.g., P53 pathway, and PI3K AKT mTOR signaling) and immune responses (e.g., apoptosis, and IL6 JAK-STAT3 signaling).

### 3.5. Stemness Subtype Differences in Immune Infiltration

**Figure 5:**
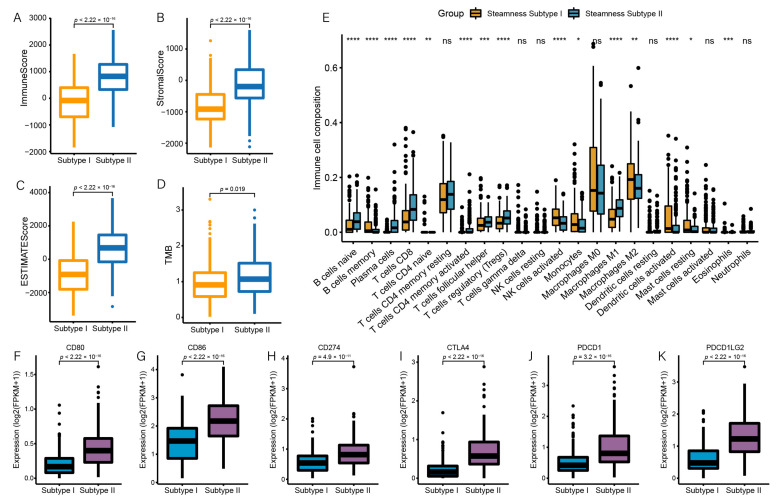
The two stemness subtypes exhibit distinct differences in immune infiltration. (**A**–**D**) Comparisons of the immune score, stromal score, ESTIMATE score, and TMB between the stemness subtypes I and II. (**E**) Comparisons of the abundances of 22 immune cells in the 2 subtypes. (**F**–**K**) The expression levels of *PDCD1*, *PDCD1LG2*, *CD274*, *CTLA4*, *CD86*, and *CD80* in the two subtypes. * *p* < 0.05; ** *p* < 0.01; *** *p* < 0.001, **** *p* < 0.0001.

Considering the association between mRNAsi and immune infiltration, we next compared differences in the immune infiltration between stemness subtypes. We found that the immune score, stromal score, and ESTIMATE score were higher in the stemness subtype II (*p*  <  0.001), indicating a high abundance of immune and stromal cells (Figure 5A–C). We also found that the TMB values were significantly higher in the stemness subtype II (*p* = 0.019; Figure 5D). In general, the higher the TMB, the more efficacious treatment with an immune checkpoint inhibitor is. Subsequently, CIBERSORT was used to quantify the abundance of the immune cell infiltration in OC. Most of the CD4+ and CD8+ T cell subsets, NK cells, and neutrophils were more enriched in the stemness subtype II. Plasma cells and resting mast cells were significantly more enriched in the stemness subtype I (Figure 5E). Among them, NK cells and T cells play an important role in killing tumor cells.

We also evaluated the expression level of six immune checkpoint genes in the two stemness subtypes. We found that the expression level of the checkpoint genes was significantly increased in the subtype II (*p* < 0.01, Figure 5F–K). The stemness subtype II exhibited significantly higher levels of expression of various immune signatures compared to the stemness subtype I. These findings suggest that the two stemness subtypes differ in their response to immunotherapy, and that the subtype II is more immunogenic and responds better to immunotherapy.

### 3.6. Construction and Validation of the Prognosis Risk Signature

**Figure 6:**
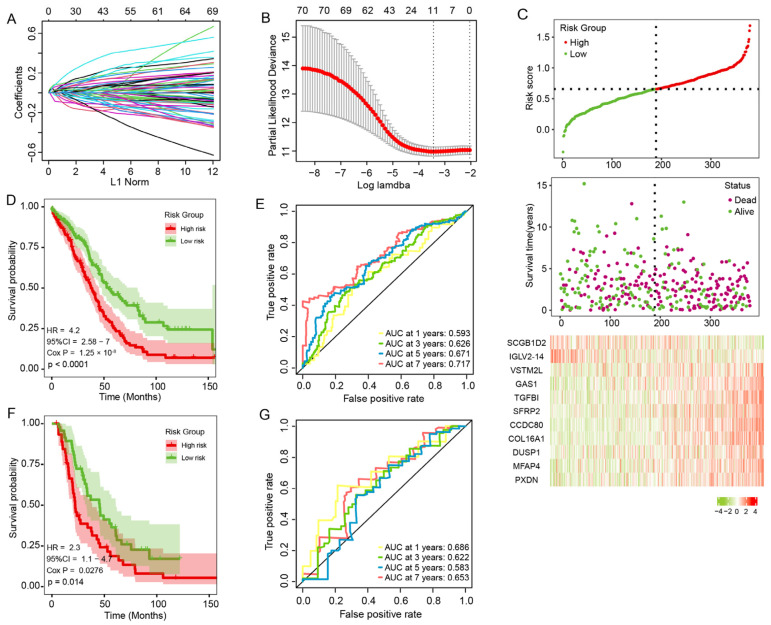
Construction and validation of the prognostic signature. (**A**,**B**) Eleven genes were identified by LASSO regression analysis. (**C**) Risk score distribution, survival status, and signature gene expression in the TCGA cohort. (**D**) The KM curves of the TCGA cohort. (**E**) The ROC curve of the TCGA cohort. (**F**) The KM curve of the ICGC cohort. (**G**) The ROC curve of the ICGC cohort.

To predict OC prognosis, we constructed an mRNAsi-related prognostic signature. Using univariate Cox regression analysis on 156 DEGs, we identified 72 genes related to OC prognosis (*p* < 0.05) and reduced this number to 11 genes using LASSO regression analysis. Then, we used these 11 genes to build a prognosis signature: *CCDC80*, *COL16A1*, *DUSP1*, *GAS1*, *IGLV2-14*, *MFAP4*, *PXDN*, *SCGB1D2*, *SFRP2*, *TGFBI*, and *VSTM2L*. Among them, *IGLV2-14* and *SCGB1D2* were associated with decreased risk with HR < 1 while the others genes were related to an increased risk with HR > 1 (Figure 6A,B). The prognostic formula was: Risk score = 0.081 × Exp (*CCDC80*) + 0.028 × Exp (*COL16A1*) + 0.01 × Exp (*DUSP1*) + 0.055 × Exp (*GAS1*) − 0.072 × Exp (*IGLV2-14*) + 0.027 × Exp (*MFAP4*) + 0.002 × Exp (*PXDN*) − 0.017 × Exp (*SCGB1D2*) + 0.003 × Exp (*SFRP2*) + 0.072 × Exp (*TGFBI*) + 0.024 × Exp (*VSTM2L*).

According to the risk formula, we counted the risk score of all patients and assigned them to low-risk (*n* = 190) or high-risk (*n* = 189) groups using the median risk score. The association between the risk score and survival information is exhibited in Figure 6C. In the TCGA cohort, the patients in the low-risk group had significantly longer overall survival times (*p* < 0.001, HR = 4.2, 95%CI: 2.58–7; Figure 6D). The AUC was 0.626 for the 3-year survival, 0.671 for the 5-year survival, and 0.717 for the 7-year survival (Figure 6E), indicating that the signature has high precision. We used the same method to assign 93 patients from ICGC to low-risk groups (*n* = 47) or high-risk groups (*n* = 46). In the ICGC cohort, patients in the low-risk group had lower death rates and longer survival times (*p* = 0.014, HR = 2.3, 95% CI: 1.1–4.7; Figure 6F). The AUC of the ICGC cohort also indicated that the model has predictive power (Figure 6G).

### 3.7. The Prognostic Signature Is an Independent Prognostic Factor for OC

**Figure 7:**
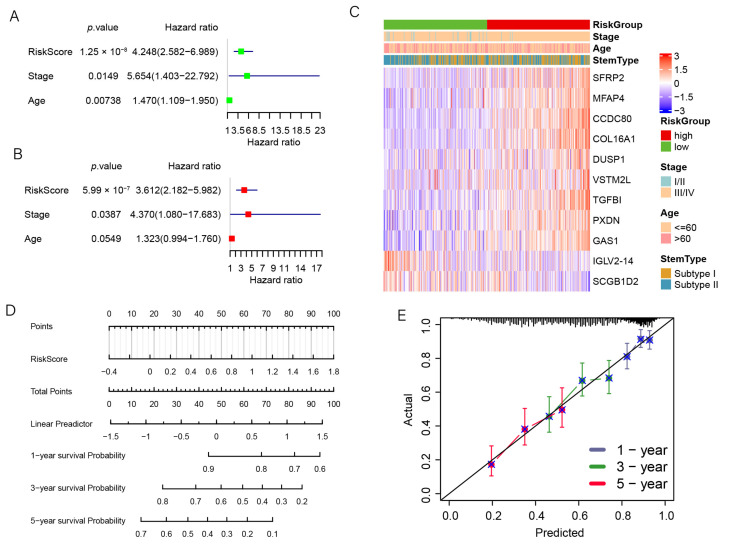
The prognostic signature was an independent prognostic factor. (**A**) The univariate Cox regression analysis. (**B**) The multivariate Cox regression analysis. (**C**) The heatmap for the connections between the clinical characteristics and the risk groups. (**D**) Nomogram of the prediction model for OC. (**E**) The nomogram calibration curves to predict the 1-, 3-, and 5-year survival.

Finally, we explored whether this signature has the ability to be independently prognostic using Cox regression analysis. The univariate Cox analysis demonstrated that the risk score and age were prognostic factors (*p* < 0.05, HR = 4.248, 95%CI: 2.582–6.989; Figure 7A), and the multivariate Cox analysis demonstrated that the risk score was an independent factor for OC (*p* < 0.05, HR = 3.612, 95%CI: 2.182–5.982; Figure 7B). We plotted the expression level of eleven genes between the two risk subgroups (Figure 7C) and found that nine genes were highly expressed in the high-risk group, suggesting that they may regulate OC progression. We established a nomogram using the prognostic signature (Figure 7D). The calibration curves for the 1-year, 3-year, and 5-year survival indicate a high degree of overlap between the actual survival rate and the survival rate predicted by the nomogram (Figure 7E). This suggests that the nomogram has a great predictive value.

## 4. Discussion

OC is one of the most serious gynecological tumors and is a global public health problem. Treatment modalities for OC mainly include surgical debulking and radiotherapy or chemotherapy [4]. Because of drug resistance and the high rate of recurrence, treatment results are unsatisfactory. The identification of reliable tumor markers will significantly impact OC treatment and prognosis. CSCs play critical roles in OC growth, metastasis, and chemoresistance [21,22]. An in-depth understanding of the molecular mechanisms of CSCs in OC would help improve clinical results.

mRNAsi has been widely used to assess the clinical prognosis and treatment of various tumors [23,24,25]. Stemness-related signatures have been revealed in different cancers, including lung squamous cell carcinoma [26], hepatocellular carcinoma [27], triple-negative breast cancer [28], and gastric cancer [29]. mRNAsi has also been used to identify prognostic biomarkers and therapeutic targets in glioma [30,31]. However, there are few studies [32,33] on the stemness index in OC. Therefore, we analyzed the stemness index’s application value in OC to improve diagnosis and treatment.

Previous studies have indicated that OC stemness is related to the tumor environment and immune cells [34]. We found a significant negative correlation between mRNAsi and the OC immune score. ssGSEA revealed that the number of immune cells was significantly reduced in patients with high mRNAsi. This indicates that high mRNAsi is closely associated with a low abundance of immune cells, suggesting that CSCs may promote OC development by attenuating immune cells’ abilities. Alex Miranda et al. [35] found an inverse correlation between stemness and immune cell infiltration in solid tumors. Other research [36,37] has indicated that CSCs suppress immune system responses and improve tumor survival.

Subsequently, we redefined the low- and high-mRNAsi groups using optimal survival thresholds. The pathway enrichment analysis found that these DEGs were closely related to biological processes such as endoderm formation and the collagen metabolic process. The DEGs were significantly enriched in multiple cancer pathways such as the PI3K-Akt and Wnt signaling pathways, indicating that CSCs regulate tumor progression in multiple ways. Using the consensus class discovery method, we classified patients into two stemness subgroups that exhibited different clinical outcomes. Compared to the stemness subtype I, patients in the stemness subtype II had a longer survival time (*p* = 0.019) and a higher enrichment of immune cells and immune infiltration. The expression level of *CD274* and the TMB value were higher in the subtype II, and TMB and *CD274* reflected patients’ sensitivity to immunotherapy [38,39,40]. Despite OC’s poor response to current immunotherapy, we can combine immunotherapy with other treatments such as chemotherapy and radiotherapy to improve treatment efficiency. According to a subtype analysis, the stemness subtype II is more sensitive to and benefits more from immunotherapy. This suggests that in patients with OC, we could choose different clinical treatments based on stemness characteristics.

In the present study, we identified 11 genes related to prognosis and constructed a prognostic risk signature. KM analysis and an ROC curve indicated that the patients in the low-risk group had significantly longer overall survival times. Furthermore, we constructed a nomogram for OC patients for potential clinical application. In this prognostic signature, *SFRP2*, *MFAP4*, *CCDC80*, *COL16A1*, *DUSP1*, *VSTM2L*, *TGFBI*, *PXDN*, and *GAS1* were highly expressed in the high-risk group, and HR > 1, suggesting that they may promote OC initiation and migration. *SFRP2* promotes metastasis and resistance to therapy in various solid tumors [41,42]. The downregulation of *SFRP2* facilitates the stemness of glioma by activating Wnt/β-catenin signaling [43]. *SFRP2* also regulates non-small-cell lung cancer metastasis via modulation of mitochondrial fission [44]. Zhao et al. verified that high levels of *MFAP4* expression predict platinum-based chemotherapy resistance and imply a poor prognosis in patients with serous OC [45]. *CCDC80* is a common tumor stemness marker used in a variety of solid tumor prognostic models [46,47,48]. Studies have shown that it helps tumor cells acquire drug resistance and immune infiltration [46]. *USP1* is one cause of drug resistance in tumors, allowing them to evade chemotherapy by modulating the p38 pathway and activating the MAPK pathway [49,50]. High *TGFBI* expression accompanies tumor resistance, and it promotes breast cancer metastasis by modulating tumor hypoxia [51]. These genes have an important influence in tumorigenesis, tumor progression, and drug resistance. However, their effect in promoting OC metastasis and drug resistance has not been fully elucidated, which requires further study.

This study also had some limitations. First, we only included 93 patients from ICGC, which is a small sample size. Second, since the two stemness subtypes have obvious differences in immune infiltration, they may show different responses to immunotherapy. However, we did not have the corresponding data to verify this. Therefore, the correlation between stemness and immunotherapy responsiveness must be validated in future clinical experiments.

## 5. Conclusions

In conclusion, we analyzed the association between mRNAsi and clinical characteristics and immune infiltration and identified two stemness-related molecular subtypes. We developed a risk signature that can effectively predict the prognosis of patients with OC, providing new insights into the precise diagnosis and prognosis for these individuals.

## Data Availability

The datasets generated and analyzed during the current study are available in the TCGA and ICGC repositories. All related scripts and supported data are available at https://github.com/YUANTTX/mRNAsi-OC (accessed on 11 June 2021).

## Supplementary Materials

The following supporting information can be downloaded at: https://www.mdpi.com/article/10.3390/genes13060993/s1, Table S1: The clinical information and clustering results; Table S2: The demographics between the two stemness subtypes.
