# Supplementary material for: The Value of the Stemness Index in Ovarian Cancer Prognosis
Source: Genes (Basel). 2022 May 31;13(6):993. doi: 10.3390/genes13060993 (PMC9222264; doi:10.3390/genes13060993)
Supplement: Supplementary file 1 [file genes-13-00993-s001.zip › Supplementary/Supplementary Table S1 The clinical information and clustering results.pdf]

**Supplementary Table S1**. The clinical information and clustering results

| Sample | Stage | Grade | Age | Residual | mRNAsi | cluster |
|---|---|---|---|---|---|---|
| TCGA_13_1407_01A | Stage IIIC | G3 | 51 | | 0.850891 | 1 |
| TCGA_24_1552_01A | Stage IIIC | G3 | 77 | 1-10 mm | 0.654834 | 1 |
| TCGA_23_1026_01B | Stage IIIC | G3 | 45 | 1-10 mm | 0.581271 | 1 |
| TCGA_61_2092_01A | Stage IIIC | G3 | 57 | No Macroscopic | 0.903482 | 2 |
| TCGA_29_1703_01A | Stage IIIC | G2 | 56 | 1-10 mm | 0.666046 | 1 |
| TCGA_13_0913_02A | Stage IIIC | G3 | 53 | No Macroscopic | 0.806825 | 2 |
| TCGA_09_1659_01B | Stage IIIC | G3 | 51 | >20 mm | 0.603128 | 1 |
| TCGA_29_1777_01A | Stage IIIC | G2 | 47 | >20 mm | 0.578199 | 2 |
| TCGA_13_0893_01B | Stage IIIC | G3 | 48 | 11-20 mm | 0.729316 | 2 |
| TCGA_24_1544_01A | Stage IIIC | G3 | 71 | 1-10 mm | 0.62042 | 1 |
| TCGA_25_1316_01A | Stage IIIC | G3 | 55 | 1-10 mm | 0.578357 | 1 |
| TCGA_OY_A56Q_01A | Stage IIA | G3 | 78 | No Macroscopic | 0.66177 | 1 |
| TCGA_29_2427_01A | Stage IIIC | G3 | 60 | >20 mm | 0.653541 | 2 |
| TCGA_23_1120_01A | Stage IIIC | G3 | 60 | 1-10 mm | 0.62894 | 1 |
| TCGA_13_1509_01A | Stage IV | G3 | 64 | 1-10 mm | 0.621448 | 2 |
| TCGA_61_2104_01A | Stage IIC | G2 | 53 | No Macroscopic | 0.608687 | 2 |
| TCGA_13_1501_01A | Stage IV | G3 | 50 | >20 mm | 0.589246 | 2 |
| TCGA_13_0924_01A | Stage IV | G3 | 45 | 1-10 mm | 0.726012 | 2 |
| TCGA_13_1410_01A | Stage IV | GX | 57 | | 0.616034 | 2 |
| TCGA_04_1343_01A | Stage IV | G3 | 72 | >20 mm | 0.57823 | 2 |
| TCGA_24_1103_01A | Stage IIIC | G3 | 50 | 1-10 mm | 0.803276 | 1 |
| TCGA_29_2425_01A | Stage IIIC | G2 | 60 | >20 mm | 0.709299 | 2 |
| TCGA_24_1413_01A | Stage IIIC | G3 | 51 | 1-10 mm | 0.610477 | 1 |
| TCGA_59_2350_01A | Stage IV | G3 | 44 | 1-10 mm | 0.833476 | 1 |
| TCGA_61_2008_02A | Stage IIC | G2 | 40 | No Macroscopic | 0.682585 | 1 |
| TCGA_24_1558_01A | Stage IIIC | G3 | 73 | 1-10 mm | 0.804769 | 1 |
| TCGA_23_1029_01B | Stage IIIC | G3 | 46 | 1-10 mm | 0.685399 | 1 |
| TCGA_23_1021_01B | Stage IV | G3 | 45 | >20 mm | 0.754236 | 1 |
| TCGA_04_1542_01A | Stage IIIB | G2 | 52 | 1-10 mm | 0.7081 | 1 |
| TCGA_13_A5FT_01A | Stage IIIC | G3 | 67 | No Macroscopic | 0.88324 | 2 |
| TCGA_25_1319_01A | Stage IIIC | G3 | 73 | 11-20 mm | 0.685908 | 1 |
| TCGA_04_1356_01A | Stage IIC | G3 | 62 | | 0.644654 | 2 |
| TCGA_WR_A838_01A | Stage IIIC | G3 | 72 | | 1 | 1 |
| TCGA_61_1738_01A | Stage IIIC | G3 | 60 | 1-10 mm | 0.756007 | 2 |
| TCGA_09_1669_01A | Stage IIIA | G3 | 54 | No Macroscopic | 0.713473 | 2 |
| TCGA_61_1918_01A | Stage IV | G3 | 45 | No Macroscopic | 0.588139 | 1 |
| TCGA_24_1842_01A | Stage IIIC | G3 | 49 | 1-10 mm | 0.768306 | 2 |
| TCGA_24_0970_01B | Stage IIIC | G3 | 63 | >20 mm | 0.589669 | 1 |
| TCGA_25_1322_01A | Stage IV | G3 | 62 | >20 mm | 0.638536 | 1 |
| TCGA_29_1781_01A | Stage IIIC | G3 | 69 | No Macroscopic | 0.802755 | 2 |

| | | | | | | |
|---|---|---|---|---|---|---|
| TCGA_24_1924_01A | Stage IIIC | G3 | 65 | 1-10 mm | 0.795073 | 2 |
| TCGA_25_1634_01A | Stage IIIC | G3 | 75 | No Macroscopic | 0.670592 | 1 |
| TCGA_31_1950_01A | Stage IIIC | G2 | 76 | 1-10 mm | 0.761012 | 2 |
| TCGA_30_1714_01A | Stage IV | G3 | 68 | 1-10 mm | 0.713736 | 1 |
| TCGA_24_2281_01A | Stage IIA | G3 | 68 | 1-10 mm | 0.73157 | 2 |
| TCGA_13_0804_01A | Stage IIIC | G3 | 73 | 1-10 mm | 0.70618 | 1 |
| TCGA_09_2044_01B | Stage IIB | G3 | 77 | No Macroscopic | 0.874052 | 2 |
| TCGA_61_2110_01A | Stage IIIC | | 56 | No Macroscopic | 0.730697 | 1 |
| TCGA_13_0916_01A | Stage IIIC | G3 | 49 | No Macroscopic | 0.822682 | 2 |
| TCGA_24_1846_01A | Stage IIIC | G3 | 45 | 1-10 mm | 0.734002 | 2 |
| TCGA_09_1667_01C | Stage IIC | G2 | 61 | No Macroscopic | 0.703957 | 2 |
| TCGA_24_0979_01A | Stage IV | G3 | 53 | >20 mm | 0.689022 | 1 |
| TCGA_24_2267_01A | Stage IIB | G3 | 58 | No Macroscopic | 0.61028 | 2 |
| TCGA_13_0765_01A | Stage IIIC | G3 | 50 | 1-10 mm | 0.678801 | 2 |
| TCGA_24_0966_01A | Stage IIIC | G3 | 78 | >20 mm | 0.708955 | 1 |
| TCGA_23_1022_01A | Stage IIIC | G3 | 67 | 1-10 mm | 0.777252 | 1 |
| TCGA_25_1312_01A | Stage IV | G3 | 69 | 1-10 mm | 0.616063 | 1 |
| TCGA_24_1419_01A | Stage IIIC | G3 | 62 | 11-20 mm | 0.654186 | 1 |
| TCGA_13_1498_01A | Stage IIIC | G3 | 73 | >20 mm | 0.657893 | 1 |
| TCGA_24_1469_01A | Stage IIIC | G3 | 71 | 1-10 mm | 0.829465 | 2 |
| TCGA_25_1877_01A | Stage IIIC | G3 | 81 | 1-10 mm | 0.846369 | 2 |
| TCGA_24_2261_01A | Stage IIIC | G3 | 76 | 11-20 mm | 0.688446 | 2 |
| TCGA_04_1514_01A | Stage IIIA | G2 | 45 | No Macroscopic | 0.632639 | 1 |
| TCGA_57_1993_01A | Stage IIIC | G3 | 56 | No Macroscopic | 0.649908 | 1 |
| TCGA_10_0933_01A | Stage IIIC | G3 | 77 | No Macroscopic | 0.79022 | 1 |
| TCGA_13_0725_01A | Stage IIIC | G3 | 44 | 1-10 mm | 0.705753 | 2 |
| TCGA_10_0936_01A | Stage IIIC | G3 | 69 | >20 mm | 0.721411 | 2 |
| TCGA_61_1910_01A | Stage IIC | G3 | 56 | 1-10 mm | 0.693418 | 1 |
| TCGA_25_2400_01A | Stage IIIC | G3 | 76 | No Macroscopic | 0.816559 | 1 |
| TCGA_04_1365_01A | Stage IIIB | G3 | 87 | | 0.804375 | 2 |
| TCGA_13_0887_01A | Stage IIIC | G3 | 42 | 1-10 mm | 0.692047 | 1 |
| TCGA_04_1651_01A | Stage IIIC | G3 | 53 | 1-10 mm | 0.723594 | 1 |
| TCGA_13_1510_01A | Stage IIIC | G3 | 62 | No Macroscopic | 0.736481 | 1 |
| TCGA_29_1696_01A | Stage IIIC | G2 | 43 | 1-10 mm | 0.65956 | 1 |
| TCGA_23_1119_01A | Stage IIIC | G3 | 64 | 1-10 mm | 0.634981 | 2 |
| TCGA_24_1416_01A | Stage IV | G3 | 34 | 1-10 mm | 0.683386 | 1 |
| TCGA_29_1697_01A | Stage IIIC | G3 | 62 | >20 mm | 0.870376 | 1 |
| TCGA_04_1536_01A | Stage IV | G3 | 60 | 1-10 mm | 0.788885 | 1 |
| TCGA_13_1489_01A | Stage IIIC | G2 | 70 | | 0.839567 | 1 |
| TCGA_13_1489_02A | Stage IIIC | G2 | 70 | | 0.780571 | 1 |
| TCGA_13_0891_01A | Stage IV | G3 | 73 | No Macroscopic | 0.665217 | 1 |
| TCGA_13_1404_01A | Stage IIIC | G3 | 48 | 1-10 mm | 0.674995 | 2 |

| | | | | | | |
|---|---|---|---|---|---|---|
| TCGA_04_1341_01A | | G3 | 85 | | 0.650663 | 2 |
| TCGA_04_1361_01A | Stage IIIB | G3 | 57 | No Macroscopic | 0.645988 | 1 |
| TCGA_24_2290_01A | Stage IIIC | G3 | 56 | 1-10 mm | 0.807045 | 2 |
| TCGA_23_1123_01A | Stage IIIC | G3 | 59 | 11-20 mm | 0.782643 | 2 |
| TCGA_24_1428_01A | Stage IIIC | G3 | 50 | 1-10 mm | 0.796488 | 2 |
| TCGA_24_1474_01A | Stage IIIC | G3 | 57 | 1-10 mm | 0.895255 | 1 |
| TCGA_13_1488_01A | Stage IV | G3 | 59 | 11-20 mm | 0.760605 | 2 |
| TCGA_30_1866_01A | Stage IV | G2 | 61 | 1-10 mm | 0.759857 | 1 |
| TCGA_61_2000_01A | Stage IIIC | G3 | 67 | No Macroscopic | 0.822084 | 1 |
| TCGA_09_2051_01A | Stage IIIC | G3 | 42 | No Macroscopic | 0.794348 | 2 |
| TCGA_24_1551_01A | Stage IIIC | G3 | 53 | 1-10 mm | 0.778244 | 2 |
| TCGA_30_1853_01A | Stage IIIC | G3 | 58 | 1-10 mm | 0.636458 | 1 |
| TCGA_09_1662_01A | Stage IV | G3 | 58 | 1-10 mm | 0.706216 | 1 |
| TCGA_24_1431_01A | Stage IIIC | G3 | 67 | 1-10 mm | 0.793344 | 1 |
| TCGA_59_2348_01A | Stage IIIC | G3 | 59 | | 0.593253 | 2 |
| TCGA_20_1686_01A | Stage IIIC | G3 | 75 | No Macroscopic | 0.791785 | 2 |
| TCGA_61_1736_01B | Stage IIIC | G3 | 45 | | 0.64435 | 1 |
| TCGA_23_1809_01A | Stage IIC | G3 | 63 | 1-10 mm | 0.666834 | 1 |
| TCGA_24_1604_01A | Stage IIIC | G3 | 66 | | 0.642813 | 2 |
| TCGA_25_2393_01A | Stage IIIC | G3 | 81 | 11-20 mm | 0.907363 | 2 |
| TCGA_61_1725_01A | Stage IIIC | G3 | 40 | 11-20 mm | 0.728861 | 2 |
| TCGA_23_2084_01A | Stage IV | G3 | 45 | No Macroscopic | 0.773123 | 1 |
| TCGA_24_1567_01A | Stage IIIB | G3 | 54 | 1-10 mm | 0.794284 | 1 |
| TCGA_5X_AA5U_01A | Stage IIC | GX | 61 | | 0.637772 | 1 |
| TCGA_61_1914_01A | Stage IIIC | G3 | 65 | >20 mm | 0.661813 | 2 |
| TCGA_24_1845_01A | Stage IIIC | G3 | 42 | 1-10 mm | 0.84423 | 1 |
| TCGA_24_1843_01A | Stage IIIC | G3 | 66 | 1-10 mm | 0.804474 | 2 |
| TCGA_29_1785_01A | Stage IIIC | G3 | 55 | >20 mm | 0.681733 | 2 |
| TCGA_04_1362_01A | Stage IIC | G3 | 59 | 1-10 mm | 0.582249 | 1 |
| TCGA_24_1426_01A | Stage IIIC | G3 | 43 | 1-10 mm | 0.625688 | 1 |
| TCGA_13_1496_01A | Stage IIIC | G3 | 65 | >20 mm | 0.819623 | 2 |
| TCGA_04_1519_01A | Stage IIIC | G3 | 48 | | 0.69559 | 1 |
| TCGA_13_0905_01B | Stage IIIC | G3 | 51 | | 0.679291 | 2 |
| TCGA_36_1570_01A | Stage IIIC | G3 | 49 | >20 mm | 0.747782 | 1 |
| TCGA_25_2399_01A | Stage IIIC | G3 | 80 | >20 mm | 0.637176 | 1 |
| TCGA_24_1471_01A | Stage IIIC | G3 | 60 | >20 mm | 0.746187 | 1 |
| TCGA_61_1741_01A | Stage IIIB | G3 | 76 | >20 mm | 0.75227 | 2 |
| TCGA_13_1512_01A | Stage IIIC | G3 | 49 | No Macroscopic | 0.687343 | 2 |
| TCGA_61_1995_01A | Stage IIIC | G3 | 43 | 1-10 mm | 0.718988 | 1 |
| TCGA_25_1627_01A | Stage IIIC | G3 | 73 | 1-10 mm | 0.700729 | 2 |
| TCGA_23_1113_01A | Stage IV | G3 | 48 | 1-10 mm | 0.695802 | 1 |
| TCGA_13_0762_01A | Stage IIIC | G3 | 65 | No Macroscopic | 0.632072 | 1 |

| | | | | | | |
|---|---|---|---|---|---|---|
| TCGA_29_1688_01A | Stage IIIC | G2 | 39 | | 0.762041 | 2 |
| TCGA_36_1577_01A | Stage IIC | G2 | 43 | No Macroscopic | 0.742971 | 1 |
| TCGA_13_1483_01A | Stage IIIC | G3 | 61 | >20 mm | 0.637853 | 1 |
| TCGA_09_2056_01B | Stage IIIC | G3 | 62 | No Macroscopic | 0.730705 | 1 |
| TCGA_24_2023_01A | Stage IIIA | G3 | 54 | 1-10 mm | 0.698393 | 1 |
| TCGA_23_2077_01A | Stage IIIC | G3 | 45 | No Macroscopic | 0.586253 | 1 |
| TCGA_24_1549_01A | Stage IIIB | G3 | 58 | | 0.689821 | 2 |
| TCGA_59_A5PD_01A | Stage IC | G3 | 55 | | 0.853351 | 2 |
| TCGA_61_1907_01A | Stage IIIC | G3 | 63 | 11-20 mm | 0.819942 | 2 |
| TCGA_24_1565_01A | Stage IIIC | G3 | 74 | 1-10 mm | 0.609265 | 1 |
| TCGA_20_1682_01A | Stage IIIC | GX | 56 | 11-20 mm | 0.590417 | 2 |
| TCGA_13_0884_01B | Stage IIIC | G3 | 39 | 1-10 mm | 0.678081 | 2 |
| TCGA_13_1507_01A | Stage IIIC | G3 | 77 | 1-10 mm | 0.588011 | 2 |
| TCGA_13_0906_01A | Stage IIIC | G3 | 50 | 1-10 mm | 0.773323 | 2 |
| TCGA_13_1409_01A | Stage IIIC | G3 | 73 | 1-10 mm | 0.682642 | 1 |
| TCGA_13_1495_01A | Stage IIIC | G2 | 60 | 1-10 mm | 0.706462 | 1 |
| TCGA_09_1670_01A | Stage IIIA | G3 | 57 | No Macroscopic | 0.764809 | 1 |
| TCGA_24_1603_01A | Stage IIIB | G3 | 53 | | 0.750118 | 1 |
| TCGA_24_2020_01A | Stage IIIC | G3 | 67 | 1-10 mm | 0.772888 | 1 |
| TCGA_13_0900_01B | Stage IIIC | G3 | 59 | | 0.609051 | 2 |
| TCGA_13_0901_01B | Stage IIIC | G3 | 41 | >20 mm | 0.71751 | 2 |
| TCGA_24_1105_01A | Stage IIIC | G3 | 36 | 1-10 mm | 0.649499 | 2 |
| TCGA_20_0991_01A | Stage IIB | G3 | 78 | No Macroscopic | 0.747703 | 2 |
| TCGA_61_1724_01A | Stage IIIC | G3 | 47 | No Macroscopic | 0.606448 | 2 |
| TCGA_24_1844_01A | Stage IIIC | G3 | 64 | 1-10 mm | 0.582132 | 1 |
| TCGA_24_1464_01A | Stage IIIC | G3 | 70 | >20 mm | 0.815323 | 1 |
| TCGA_24_1616_01A | Stage IIIC | G3 | 56 | 1-10 mm | 0.790385 | 1 |
| TCGA_04_1364_01A | Stage IIIC | G3 | 61 | 1-10 mm | 0.917715 | 1 |
| TCGA_61_1911_01A | Stage IIA | G3 | 55 | | 0.630067 | 2 |
| TCGA_VG_A8LO_01A | Stage IV | GB | 55 | >20 mm | 0.737666 | 1 |
| TCGA_23_1027_01A | Stage IIIC | G3 | 48 | 1-10 mm | 0.645246 | 1 |
| TCGA_10_0931_01A | Stage IIIC | G3 | 44 | 1-10 mm | 0.872442 | 1 |
| TCGA_13_0795_01A | Stage IIIC | G3 | 66 | >20 mm | 0.715264 | 2 |
| TCGA_04_1347_01A | Stage IV | G3 | 81 | No Macroscopic | 0.749413 | 2 |
| TCGA_04_1655_01A | Stage IIIB | G2 | 49 | No Macroscopic | 0.616142 | 1 |
| TCGA_59_2351_01A | Stage IIIC | G3 | 51 | 1-10 mm | 0.72952 | 2 |
| TCGA_29_1774_01A | Stage IIIC | G3 | 82 | >20 mm | 0.672099 | 1 |
| TCGA_61_2111_01A | Stage IV | G3 | 61 | | 0.577859 | 2 |
| TCGA_24_0982_01A | Stage IIIC | G3 | 77 | 1-10 mm | 0.676725 | 2 |
| TCGA_31_1944_01A | Stage IIIC | G3 | 47 | No Macroscopic | 0.5915 | 2 |
| TCGA_13_2060_01A | Stage IV | G3 | 51 | 1-10 mm | 0.821152 | 2 |
| TCGA_24_2026_01A | Stage IIIC | G3 | 79 | >20 mm | 0.667075 | 2 |

| | | | | | | |
|---|---|---|---|---|---|---|
| TCGA_13_0766_01A | Stage IIIC | G3 | 42 | 1-10 mm | 0.841386 | 1 |
| TCGA_24_2033_01A | Stage IIIC | G3 | 87 | >20 mm | 0.597732 | 1 |
| TCGA_09_2053_01C | Stage IIIC | G3 | 72 | No Macroscopic | 0.675266 | 2 |
| TCGA_24_1557_01A | Stage IIIC | G3 | 49 | >20 mm | 0.717265 | 1 |
| TCGA_30_1718_01A | Stage IIIC | G3 | 44 | 1-10 mm | 0.604201 | 2 |
| TCGA_36_1571_01A | Stage IIIB | G3 | 53 | No Macroscopic | 0.757369 | 1 |
| TCGA_13_0897_01A | Stage IIIC | G3 | 54 | 1-10 mm | 0.6069 | 1 |
| TCGA_29_1701_01A | Stage IIIC | G3 | 56 | | 0.781813 | 2 |
| TCGA_13_0797_01A | Stage IIIC | G3 | 49 | 1-10 mm | 0.812661 | 2 |
| TCGA_25_1315_01A | Stage IIIC | G3 | 50 | 1-10 mm | 0.711542 | 1 |
| TCGA_61_1900_01A | Stage IIIB | G3 | 51 | No Macroscopic | 0.681114 | 1 |
| TCGA_24_1467_01A | Stage IIIC | G3 | 51 | 1-10 mm | 0.640045 | 1 |
| TCGA_09_0367_01A | Stage IIIC | G3 | 67 | 1-10 mm | 0.666839 | 1 |
| TCGA_13_1506_01A | Stage IIIC | G3 | 45 | | 0.964705 | 2 |
| TCGA_10_0937_01A | Stage IIIC | G3 | 44 | 1-10 mm | 0.765236 | 1 |
| TCGA_23_1024_01A | Stage IV | G3 | 52 | 1-10 mm | 0.879532 | 1 |
| TCGA_23_1110_01A | Stage IIIC | G3 | 42 | No Macroscopic | 0.772278 | 1 |
| TCGA_13_0920_01A | Stage IIIC | G3 | 65 | 1-10 mm | 0.799481 | 2 |
| TCGA_25_2396_01A | Stage IIIC | G3 | 71 | No Macroscopic | 0.724869 | 2 |
| TCGA_20_0987_01A | Stage IIIC | G3 | 61 | 1-10 mm | 0.714472 | 2 |
| TCGA_29_1769_01A | Stage IIIC | G3 | 40 | 1-10 mm | 0.724793 | 2 |
| TCGA_10_0938_01A | Stage IIIC | G3 | 80 | >20 mm | 0.841656 | 1 |
| TCGA_10_0928_01A | Stage IIIC | G3 | 71 | 1-10 mm | 0.744102 | 1 |
| TCGA_13_1497_01A | Stage IIIC | G3 | 47 | | 0.695685 | 2 |
| TCGA_24_1417_01A | Stage IV | G3 | 54 | 1-10 mm | 0.640286 | 2 |
| TCGA_59_2363_01A | Stage IIIA | G3 | 40 | No Macroscopic | 0.721077 | 1 |
| TCGA_13_1492_01A | Stage IIIC | G3 | 66 | | 0.881514 | 1 |
| TCGA_29_1762_01A | Stage IV | G2 | 59 | >20 mm | 0.842862 | 1 |
| TCGA_20_1683_01A | Stage IIIC | G3 | 65 | No Macroscopic | 0.57828 | 2 |
| TCGA_13_0800_01A | Stage IIIC | G3 | 52 | No Macroscopic | 0.707728 | 1 |
| TCGA_57_1584_01A | Stage IIIC | G3 | 47 | 1-10 mm | 0.692477 | 1 |
| TCGA_24_1850_01A | Stage IIIC | G3 | 72 | 1-10 mm | 0.618303 | 2 |
| TCGA_29_2414_02A | Stage IIIC | G2 | 75 | 11-20 mm | 0.713778 | 2 |
| TCGA_23_1107_01A | Stage IV | G3 | 59 | >20 mm | 0.906042 | 2 |
| TCGA_23_1028_01A | Stage IIIC | G3 | 43 | 1-10 mm | 0.799083 | 2 |
| TCGA_29_1778_01A | Stage IIIC | G3 | 77 | No Macroscopic | 0.583673 | 2 |
| TCGA_13_0724_01A | Stage IV | G3 | 72 | 1-10 mm | 0.678564 | 2 |
| TCGA_09_0364_01A | Stage IIC | G3 | 80 | 11-20 mm | 0.778091 | 1 |
| TCGA_29_1784_01A | Stage IIIC | G3 | 55 | No Macroscopic | 0.783359 | 2 |
| TCGA_29_1691_01A | Stage IIIC | G2 | 51 | 1-10 mm | 0.797236 | 2 |
| TCGA_29_1761_01A | Stage IIIC | G3 | 80 | >20 mm | 0.945825 | 2 |
| TCGA_57_1582_01A | Stage IIIC | G3 | 50 | 1-10 mm | 0.796474 | 2 |

| | | | | | | |
|---|---|---|---|---|---|---|
| TCGA_04_1350_01A | Stage IIIC | G3 | 46 | >20 mm | 0.721473 | 1 |
| TCGA_24_2027_01A | Stage IV | G3 | 51 | >20 mm | 0.655313 | 1 |
| TCGA_23_1122_01A | Stage IIIC | G3 | 53 | 1-10 mm | 0.634357 | 1 |
| TCGA_09_2048_01A | Stage IIIC | G3 | 63 | 1-10 mm | 0.68237 | 1 |
| TCGA_57_1586_01A | Stage IIIC | G3 | 66 | 11-20 mm | 0.637482 | 2 |
| TCGA_13_0885_01A | Stage IIIC | G3 | 70 | 11-20 mm | 0.827442 | 2 |
| TCGA_29_1707_02A | Stage IIC | G3 | 41 | No Macroscopic | 0.731499 | 1 |
| TCGA_61_2109_01A | Stage IIIC | G3 | 40 | >20 mm | 0.715057 | 1 |
| TCGA_24_1560_01A | Stage IIIC | G4 | 51 | 1-10 mm | 0.732634 | 1 |
| TCGA_24_1470_01A | Stage IIIB | G3 | 54 | 1-10 mm | 0.610492 | 2 |
| TCGA_23_1118_01A | Stage IIIC | G3 | 45 | >20 mm | 0.826017 | 2 |
| TCGA_57_1583_01A | Stage IIIC | G3 | 57 | No Macroscopic | 0.802708 | 1 |
| TCGA_25_1630_01A | Stage IIIC | G3 | 73 | 1-10 mm | 0.608061 | 1 |
| TCGA_36_1581_01A | Stage IIC | G3 | 63 | No Macroscopic | 0.705605 | 2 |
| TCGA_36_1574_01A | Stage IIIC | G3 | 48 | >20 mm | 0.708284 | 2 |
| TCGA_23_1030_01A | Stage IIIC | G3 | 64 | 1-10 mm | 0.599465 | 1 |
| TCGA_09_1665_01B | Stage IIIC | G2 | 73 | No Macroscopic | 0.735808 | 2 |
| TCGA_13_0923_01A | Stage IIIC | G3 | 74 | No Macroscopic | 0.804361 | 1 |
| TCGA_25_2391_01A | Stage IIIC | G3 | 57 | >20 mm | 0.707328 | 1 |
| TCGA_29_2428_01A | Stage IIIC | G3 | 58 | >20 mm | 0.598987 | 2 |
| TCGA_24_0968_01A | Stage IIIC | G3 | 59 | 1-10 mm | 0.636877 | 2 |
| TCGA_09_1666_01A | Stage IIIC | G3 | 57 | No Macroscopic | 0.677968 | 1 |
| TCGA_30_1860_01A | Stage IIIC | G3 | 58 | >20 mm | 0.777698 | 2 |
| TCGA_30_1857_01A | Stage IV | G3 | 64 | | 0.425025 | 2 |
| TCGA_24_1422_01A | Stage IIIC | G3 | 82 | 1-10 mm | 0.440547 | 2 |
| TCGA_61_1737_01A | Stage IV | G3 | 42 | 1-10 mm | 0.576053 | 1 |
| TCGA_13_0768_01A | Stage IIIC | G3 | 73 | 1-10 mm | 0.38838 | 2 |
| TCGA_13_1411_01A | Stage IIIC | G3 | 81 | >20 mm | 0.41906 | 2 |
| TCGA_13_1408_01A | Stage IIIC | G3 | 59 | >20 mm | 0.392464 | 2 |
| TCGA_61_2102_01A | Stage IIIC | G3 | 74 | | 0.334383 | 1 |
| TCGA_29_1695_01A | Stage IIIC | G2 | 62 | >20 mm | 0.563089 | 2 |
| TCGA_29_1694_01A | Stage IIIC | GX | 45 | 11-20 mm | 0.454938 | 2 |
| TCGA_23_1023_01A | Stage IIIC | G3 | 65 | 1-10 mm | 0.483398 | 1 |
| TCGA_13_1405_01A | Stage IV | G3 | 49 | 1-10 mm | 0.453494 | 2 |
| TCGA_09_0366_01A | Stage IIIC | G3 | 55 | 1-10 mm | 0.464586 | 1 |
| TCGA_24_1553_01A | Stage IIIB | G3 | 53 | 1-10 mm | 0.519142 | 2 |
| TCGA_23_1109_01A | Stage IIIC | G3 | 62 | 1-10 mm | 0.555921 | 2 |
| TCGA_24_2035_01A | Stage IIIC | G3 | 65 | 1-10 mm | 0.269951 | 2 |
| TCGA_61_2008_01A | Stage IIC | G2 | 40 | No Macroscopic | 0.571574 | 2 |
| TCGA_61_2012_01A | Stage IIC | G2 | 81 | | 0.556371 | 2 |
| TCGA_29_1690_01A | Stage IIIC | G2 | 66 | 11-20 mm | 0.339335 | 1 |
| TCGA_29_1763_01A | Stage IIC | G2 | 43 | 1-10 mm | 0.436271 | 1 |

| | | | | | | |
|---|---|---|---|---|---|---|
| TCGA_23_2078_01A | Stage IIIC | G3 | 66 | No Macroscopic | 0.460538 | 2 |
| TCGA_25_1329_01A | Stage IIIC | G3 | 76 | 11-20 mm | 0.49294 | 2 |
| TCGA_25_1635_01A | Stage IIIC | G3 | 71 | 1-10 mm | 0.507227 | 2 |
| TCGA_36_1580_01A | Stage IIIC | G3 | 82 | >20 mm | 0.388454 | 2 |
| TCGA_25_2404_01A | Stage IIIC | G3 | 38 | No Macroscopic | 0.423464 | 2 |
| TCGA_36_1569_01A | Stage IIIC | G3 | 52 | 1-10 mm | 0.41167 | 2 |
| TCGA_25_1328_01A | Stage IIIC | G3 | 38 | 1-10 mm | 0.240866 | 2 |
| TCGA_13_1505_01A | Stage IIIC | G3 | 63 | 1-10 mm | 0.466528 | 2 |
| TCGA_20_1687_01A | Stage IV | G3 | 46 | No Macroscopic | 0.525044 | 2 |
| TCGA_25_1323_01A | Stage IIIC | G3 | 72 | 1-10 mm | 0.576929 | 1 |
| TCGA_13_1477_01A | Stage IV | G2 | 49 | 1-10 mm | 0.455264 | 1 |
| TCGA_31_1959_01A | Stage IV | G2 | 49 | | 0.413524 | 2 |
| TCGA_25_2398_01A | Stage IIIC | G3 | 71 | 1-10 mm | 0.316201 | 1 |
| TCGA_13_0714_01A | Stage IV | G3 | 55 | 1-10 mm | 0.542156 | 2 |
| TCGA_36_1576_01A | Stage IIIC | G3 | 76 | >20 mm | 0.401148 | 2 |
| TCGA_24_2280_01A | Stage IIIC | G3 | 74 | 1-10 mm | 0.248386 | 2 |
| TCGA_25_1326_01A | Stage IIIC | G3 | 61 | 1-10 mm | 0.517196 | 2 |
| TCGA_29_1711_01A | Stage IIIC | G2 | 45 | No Macroscopic | 0.558911 | 2 |
| TCGA_09_1673_01A | Stage IV | G3 | 50 | >20 mm | 0.54227 | 1 |
| TCGA_29_1783_01A | Stage IIIC | G3 | 58 | 1-10 mm | 0.465988 | 2 |
| TCGA_61_2003_01A | Stage IIIC | G2 | 53 | 1-10 mm | 0.328291 | 1 |
| TCGA_24_1847_01A | Stage IV | G3 | 45 | 1-10 mm | 0.505673 | 2 |
| TCGA_25_1313_01A | Stage IV | G3 | 62 | 1-10 mm | 0.473055 | 1 |
| TCGA_25_2401_01A | Stage IIIC | G3 | 64 | 1-10 mm | 0.543146 | 2 |
| TCGA_61_1733_01A | Stage IIIC | G3 | 71 | No Macroscopic | 0.451458 | 1 |
| TCGA_57_1585_01A | Stage IIIC | G3 | 57 | 11-20 mm | 0.356493 | 2 |
| TCGA_25_1632_01A | Stage IV | G3 | 68 | 1-10 mm | 0.497284 | 1 |
| TCGA_25_1321_01A | Stage IIIC | G3 | 65 | No Macroscopic | 0.570972 | 1 |
| TCGA_24_1104_01A | Stage IV | G3 | 56 | >20 mm | 0.496317 | 2 |
| TCGA_25_2392_01A | Stage IV | G3 | 75 | 11-20 mm | 0.562175 | 2 |
| TCGA_24_1928_01A | Stage IIIC | G3 | 77 | 11-20 mm | 0.481006 | 1 |
| TCGA_25_2409_01A | Stage IV | G3 | 71 | 1-10 mm | 0.461665 | 2 |
| TCGA_61_2098_01A | Stage IIIC | G2 | 62 | 11-20 mm | 0.487704 | 1 |
| TCGA_04_1530_01A | Stage IIIC | G3 | 68 | 1-10 mm | 0.499837 | 2 |
| TCGA_13_0730_01A | Stage IIIC | G3 | 71 | >20 mm | 0.543144 | 1 |
| TCGA_59_2355_01A | Stage IV | G3 | 58 | >20 mm | 0.545948 | 1 |
| TCGA_24_1418_01A | Stage IIIC | G3 | 68 | >20 mm | 0.533549 | 2 |
| TCGA_30_1862_01A | Stage IV | G2 | 65 | >20 mm | 0.454277 | 2 |
| TCGA_24_2293_01A | | G3 | 47 | >20 mm | 0.246019 | 2 |
| TCGA_61_2009_01A | Stage IIIC | G3 | 65 | 11-20 mm | 0.479734 | 2 |
| TCGA_29_1770_01A | Stage IIIC | G2 | 54 | 1-10 mm | 0.503631 | 1 |
| TCGA_59_2354_01A | Stage IIIC | G3 | 63 | 1-10 mm | 0.409559 | 2 |

| | | | | | | |
|---|---|---|---|---|---|---|
| TCGA_24_2288_01A | Stage IIIC | G3 | 70 | >20 mm | 0.520144 | 2 |
| TCGA_09_2045_01A | Stage IV | G2 | 50 | 1-10 mm | 0.478551 | 1 |
| TCGA_04_1357_01A | Stage IIIB | G3 | 52 | | 0.548003 | 2 |
| TCGA_29_1693_01A | Stage IIIC | G3 | 72 | >20 mm | 0.526596 | 2 |
| TCGA_09_0369_01A | Stage IIIC | G3 | 56 | No Macroscopic | 0.567278 | 1 |
| TCGA_24_1550_01A | Stage IIIC | G3 | 49 | 1-10 mm | 0.308327 | 2 |
| TCGA_31_1956_01A | Stage IIIB | G3 | 60 | | 0.494963 | 2 |
| TCGA_30_1891_01A | Stage IIIC | G2 | 61 | 1-10 mm | 0.467565 | 2 |
| TCGA_09_1661_01B | Stage IIIC | G3 | 75 | 1-10 mm | 0.48618 | 1 |
| TCGA_30_1892_01A | Stage IIIC | G3 | 52 | 1-10 mm | 0.30937 | 2 |
| TCGA_24_2297_01A | Stage IIIC | G3 | 56 | >20 mm | 0.550876 | 1 |
| TCGA_59_2352_01A | Stage IIIC | G3 | 78 | | 0.46747 | 2 |
| TCGA_29_1705_01A | Stage IIIC | G2 | 47 | >20 mm | 0.559757 | 1 |
| TCGA_13_1485_01A | Stage IV | G2 | 48 | 1-10 mm | 0.14474 | 1 |
| TCGA_29_1768_01A | Stage IV | G3 | 50 | >20 mm | 0.464434 | 2 |
| TCGA_24_1546_01A | Stage IIIC | G3 | 46 | 1-10 mm | 0.314051 | 2 |
| TCGA_24_1430_01A | Stage IIIC | G3 | 68 | >20 mm | 0.566898 | 1 |
| TCGA_24_2271_01A | Stage IIIC | G3 | 55 | >20 mm | 0.476237 | 2 |
| TCGA_29_1776_01A | Stage IIIC | G3 | 63 | >20 mm | 0.539167 | 2 |
| TCGA_24_1425_01A | Stage IIIC | G3 | 45 | 1-10 mm | 0.329697 | 2 |
| TCGA_13_0883_01A | Stage IIIC | G3 | 61 | 1-10 mm | 0.340954 | 1 |
| TCGA_13_0888_01A | Stage IIIC | G3 | 78 | No Macroscopic | 0.484268 | 1 |
| TCGA_29_A5NZ_01A | Stage IIIC | G3 | 66 | | 0.44377 | 2 |
| TCGA_57_1994_01A | | | 63 | | 0.569476 | 2 |
| TCGA_61_1728_01A | Stage IV | G3 | 59 | 1-10 mm | 0.567876 | 1 |
| TCGA_61_2113_01A | Stage IIC | G3 | 53 | | 0.499945 | 2 |
| TCGA_61_2097_01A | Stage IIC | G2 | 71 | 1-10 mm | 0.387556 | 1 |
| TCGA_10_0927_01A | Stage IIIC | G2 | 65 | 1-10 mm | 0.515576 | 2 |
| TCGA_13_0908_01B | Stage IV | G3 | 58 | 1-10 mm | 0.381871 | 2 |
| TCGA_31_1953_01A | Stage IIIC | G3 | 52 | | 0.527836 | 2 |
| TCGA_24_1923_01A | Stage IIIC | G3 | 51 | 1-10 mm | 0.450786 | 1 |
| TCGA_29_1710_01A | Stage IIIC | G2 | 54 | 1-10 mm | 0.425275 | 2 |
| TCGA_24_2289_01A | Stage IV | G3 | 68 | >20 mm | 0.379855 | 1 |
| TCGA_61_1721_01A | Stage IV | G1 | 38 | | 0 | 1 |
| TCGA_30_1861_01A | Stage IIIC | G3 | 74 | 1-10 mm | 0.430111 | 1 |
| TCGA_04_1648_01A | Stage IIIC | G2 | 57 | 1-10 mm | 0.534768 | 1 |
| TCGA_25_1631_01A | Stage IIIC | G3 | 73 | 1-10 mm | 0.577426 | 1 |
| TCGA_13_1487_01A | Stage IV | GX | 74 | | 0.5007 | 1 |
| TCGA_24_2038_01A | Stage IIIA | GB | 68 | 1-10 mm | 0.387699 | 1 |
| TCGA_31_1946_01A | Stage IIIC | G3 | 30 | No Macroscopic | 0.540859 | 2 |
| TCGA_13_1511_01A | Stage IV | G3 | 52 | >20 mm | 0.556702 | 1 |
| TCGA_23_1116_01A | Stage IIIC | G3 | 83 | >20 mm | 0.421906 | 2 |

| | | | | | | |
|---|---|---|---|---|---|---|
| TCGA_25_1320_01A | Stage IIIC | G3 | 65 | 1-10 mm | 0.463068 | 2 |
| TCGA_23_1111_01A | Stage IIIC | G3 | 63 | >20 mm | 0.50514 | 1 |
| TCGA_13_0726_01A | Stage IIIC | GX | 55 | 1-10 mm | 0.465502 | 1 |
| TCGA_25_1317_01A | Stage IIIC | G3 | 66 | | 0.491851 | 2 |
| TCGA_25_1623_01A | Stage IV | G3 | 71 | No Macroscopic | 0.479769 | 2 |
| TCGA_04_1331_01A | Stage IIIC | G3 | 78 | 1-10 mm | 0.557884 | 2 |
| TCGA_23_1114_01B | Stage IIIC | G3 | 55 | No Macroscopic | 0.416233 | 1 |
| TCGA_25_1626_01A | Stage IIIC | G3 | 65 | >20 mm | 0.175682 | 2 |
| TCGA_25_1870_01A | Stage IIIC | G3 | 59 | 11-20 mm | 0.527021 | 1 |
| TCGA_09_2054_01A | Stage IIIC | G3 | 58 | 1-10 mm | 0.41538 | 2 |
| TCGA_24_2036_01A | Stage IIIA | G3 | 50 | 1-10 mm | 0.56528 | 1 |
| TCGA_04_1338_01A | Stage IIIC | G3 | 78 | 1-10 mm | 0.448185 | 2 |
| TCGA_24_1424_01A | Stage IIIC | G3 | 67 | 1-10 mm | 0.55169 | 1 |
| TCGA_29_1766_01A | Stage IIIC | G2 | 74 | 11-20 mm | 0.558555 | 2 |
| TCGA_13_0720_01A | Stage IIIC | G3 | 48 | 1-10 mm | 0.559624 | 2 |
| TCGA_24_2262_01A | Stage IIIC | G3 | 57 | 1-10 mm | 0.489905 | 2 |
| TCGA_24_2254_01A | Stage IIIC | G3 | 66 | 1-10 mm | 0.368783 | 1 |
| TCGA_24_1930_01A | Stage IIIC | G3 | 53 | 1-10 mm | 0.46236 | 2 |
| TCGA_24_1427_01A | Stage IIIC | G3 | 58 | >20 mm | 0.384058 | 2 |
| TCGA_29_2414_01A | Stage IIIC | G2 | 75 | 11-20 mm | 0.528913 | 2 |
| TCGA_24_2024_01A | Stage IIIC | G3 | 72 | 1-10 mm | 0.448733 | 2 |
| TCGA_24_2298_01A | Stage IIIC | G3 | 55 | >20 mm | 0.442456 | 2 |
| TCGA_25_2042_01A | Stage IIIC | G3 | 60 | >20 mm | 0.093629 | 2 |
| TCGA_61_1919_01A | Stage IIIC | G2 | 58 | | 0.556965 | 2 |
| TCGA_25_1633_01A | Stage IIIC | G3 | 64 | 1-10 mm | 0.306656 | 2 |
| TCGA_13_0727_01A | Stage IIIC | G3 | 71 | 1-10 mm | 0.562614 | 1 |
| TCGA_24_1562_01A | Stage IIIC | G3 | 67 | 1-10 mm | 0.565576 | 2 |
| TCGA_24_1563_01A | Stage IIIC | G3 | 66 | 1-10 mm | 0.425447 | 1 |
| TCGA_31_1951_01A | Stage IIIC | G3 | 58 | No Macroscopic | 0.458671 | 2 |
| TCGA_36_1568_01A | Stage IIIC | G3 | 52 | | 0.525945 | 2 |
| TCGA_13_0886_01A | Stage IIIC | G3 | 67 | 1-10 mm | 0.513498 | 2 |
| TCGA_61_1998_01A | Stage IIIC | G3 | 48 | 1-10 mm | 0.492209 | 1 |
| TCGA_04_1332_01A | Stage IIIC | G3 | 70 | 1-10 mm | 0.285667 | 1 |
| TCGA_61_2088_01A | Stage IIIC | G3 | 51 | No Macroscopic | 0.421798 | 1 |
| TCGA_24_1434_01A | Stage IIIC | G3 | 59 | 1-10 mm | 0.298204 | 2 |
| TCGA_25_1628_01A | Stage IIIC | G3 | 67 | 1-10 mm | 0.427322 | 2 |
| TCGA_24_1423_01A | Stage IIIC | G3 | 61 | 1-10 mm | 0.500199 | 1 |
| TCGA_24_1435_01A | Stage IIIC | G3 | 57 | 1-10 mm | 0.503347 | 1 |
| TCGA_13_0911_01A | Stage IV | G3 | 55 | 1-10 mm | 0.521662 | 2 |
| TCGA_61_2101_01A | Stage IIIC | G2 | 55 | 11-20 mm | 0.460626 | 1 |
| TCGA_25_1318_01A | Stage IIIC | G3 | 54 | 1-10 mm | 0.457258 | 1 |
| TCGA_13_1499_01A | Stage IIIC | G3 | 56 | | 0.390071 | 2 |

| | | | | | | |
|---|---|---|---|---|---|---|
| TCGA_09_1668_01B | Stage IIIC | G3 | 57 | | 0.556857 | 2 |
| TCGA_61_2002_01A | Stage IIIC | G3 | 46 | 11-20 mm | 0.543058 | 2 |
| TCGA_13_1403_01A | Stage IIIC | G3 | 48 | | 0.525012 | 1 |
