# Supplementary material for: The Value of the Stemness Index in Ovarian Cancer Prognosis
Source: Genes (Basel). 2022 May 31;13(6):993. doi: 10.3390/genes13060993 (PMC9222264; doi:10.3390/genes13060993)
Supplement: Supplementary file 1 [file genes-13-00993-s001.zip › Supplementary/Supplementary Table S2 The demographics between the two stemness subtypes.pdf]

Supplementary Table S2. The demographics between the two stemness subtypes

	level	Subtype I	Subtype II	p
N		178	201	
Age (%)	<=60	101 (56.4)	107 (53.5)	0.64
	>60	77 (43.6)	94 (46.5)	
OS (%)	Alive	54 (30.7)	93 (46.0)	0.003
	Dead	124 (69.3)	108 (54.0)	
Stage (%)	I	0 (0.0)	1 (0.5)	0.548
	II	11 (6.1)	12 (6.1)	
	III	136 (76.5)	159 (80.2)	
	IV	31 (17.3)	26 (13.2)	
Tumor Residual (%)	>20 mm	32 (17.9)	38 (19.0)	0.017
	1-10 mm	90 (50.8)	81 (40.0)	
	11-20 mm	8 (4.5)	19 (9.5)	
	No Macroscopic	35 (19.6)	32 (16.0)	
	NA	13 (7.3)	31 (15.5)	
Therapy outcome (%)	Complete Remission/Response	99 (55.3)	114 (57.0)	0.897
	Partial Remission/Response	20 (11.7)	23 (11.0)	
	Progressive Disease	11 (6.1)	16 (8.0)	
	Stable Disease	12 (6.7)	10 (5.0)	
	NA	36 (20.1)	38 (19.0)	
